# Climate change and COVID-19: global challenges and opportunities

**DOI:** 10.1016/j.eclinm.2021.100738

**Published:** 2021-01-28

2020 was undoubtedly dominated by the COVID-19 pandemic, which has resulted in the death of more than 2 million people worldwide thus far. The year was also marked by the increasingly evident effects of climate change, including record-breaking wildfires, flooding, and more extreme temperatures. A striking parallel between these two crises is that they both resulted in loss of life that could have been prevented through global efforts. Additionally, the pandemic may further strain livelihoods and resources already affected by climate change, requiring swift action to avoid catastrophic risks to human health.

Political and social focus has understandably shifted to mitigating the health and economic effects of the pandemic; however, climate change and its consequences should not be neglected. Climate change is particularly important because, according to the UN Environment Programme's Emissions Gap Report 2020 published in December, 2020, the planet is heading for a global temperature rise in excess of 3°C this century. This increase is a sobering thought, because December, 2020, also marked the 5-year anniversary of the signing of the Paris Agreement, which aims to limit global warming to below 2°C, compared with preindustrial levels. In the same month, *The Lancet* published *The Lancet* Countdown on Health and Climate Change*,* which emphasised that climate change will have a severe effect on our health, and consequently further strain health-care systems. The report highlights the urgency with which we need to address climate change, because the window of opportunity for lasting effects is narrowing every day. Promising changes are commitments to climate action from high-emitting countries such as China and the USA, as US President Joe Biden rejoined the Paris Agreement. Furthermore, the UN Climate Change Conference of the Parties, to be held in Glasgow in November, 2021, will accelerate action towards achieving the goals of the Paris Agreement and the UN Framework Convention on Climate Change.

Strategies to curtail the effects of the pandemic, such as lockdown and physical distancing, have significantly reduced travel, as well as the consumption of goods and services. During the initial lockdowns in Spring, 2020, global pollution levels were reported to be unusually low. A surge in emissions was observed post lockdown and, as shown in a study by Zhu Liu and colleagues (Tsinghua University, China) published in *Nature Communications*, emissions in several countries had nearly recovered by the end of June, 2020. Consequently, the UN Environment Programme's Emissions Gap Report 2020 stated that the brief reduction in emissions is expected to have a negligible long-term impact on climate change. With the distribution of coronavirus vaccines now underway, economic activity is expected to pick up again, but without meaningful changes in policies, emissions of pollutants are likely to rise again.

A noticeable response to COVID-19 restrictions worldwide was the increase in the use of parks and other green spaces for exercise and leisure. As such, the pandemic has made many more of us aware of the importance of outdoor spaces to support our health and wellbeing. This shift led to several cities around the world to announce measures to create safer environments—from new cycle lanes in Milan and London to widening pavements and pedestrianising neighbourhoods in New York. These initiatives will allow people to commute without the use of public transport system during the pandemic, and will ensure that cities can emerge from the pandemic as more sustainable and recreational places.

A report by the European Environment Agency, published in August, 2020, revealed that one in every eight deaths in Europe can be attributed to pollution. Furthermore, the report highlighted that, like COVID-19, poorer communities and vulnerable people are the most affected. Children are particularly susceptible to the harmful effects of pollution, and a 2019 study by Pattanun Achakulwisut and colleagues (George Washington University, USA) published in *The Lancet Planetary Health*, estimated that 4 million cases of childhood asthma globally could be linked to traffic-related air pollution. The severity of the issue was brought to the forefront of media attention in December, 2020, when, in a landmark case, a UK coroner ruled that air pollution was the cause of death of a 9-year-old girl from London, who suffered from severe asthma.

The substantial benefits of reducing air pollution exposure are particularly relevant in the context of the pandemic. A study by Andrea Pozzer and colleagues (Max Planck Institute for Chemistry, Germany), published in *Cardiovascular Research* in December, 2020, suggested that air pollution increases the risk of mortality from COVID-19. The pandemic has taken a disproportionate toll on specific groups of society, with ethnic minorities and people with underlying conditions facing the poorest health outcomes.

It has become evident that the COVID-19 pandemic and climate change increase barriers for improving health and widen inequalities. However, the pandemic has also shown us that unparalleled breakthroughs in health and science are possible, with the design, approval, and production of several effective vaccines less than 1 year after identification of the virus. The pandemic has also highlighted that global challenges require global solutions. As governments set out economic recovery plans for the COVID-19 aftermath, there is an opportunity to improve public health, and work towards a more equitable and sustainable future for all.***EClinicalMedicine***

